# Induction of Specific Humoral and Cellular Immune Responses in a Mouse Model following Gene Fusion of HSP70C and Hantaan Virus Gn and S0.7 in an Adenoviral Vector

**DOI:** 10.1371/journal.pone.0088183

**Published:** 2014-02-04

**Authors:** Linfeng Cheng, Lan Yu, Xingan Wu, Kai Li, Fang Wang, Liang Zhang, Wei Ye, Puyuan Li, Fanglin Zhang, Zhikai Xu

**Affiliations:** Department of Microbiology, Fourth Military Medical University, Xi’an, China; Federal University of São Paulo, Brazil

## Abstract

Heat shock proteins (HSPs) display adjuvant functions when given as fusion proteins to enhance vaccination efficiency. To evaluate enhanced potency of Hantaan virus (HTNV) glycoprotein (GP) and nucleocapsid protein (NP) immunogenicity by heat shock protein 70 (HSP70), a recombinant adenovirus rAd-GnS0.7-pCAG-HSP70C expression vector was developed by genetically linking the HSP70 C-terminal gene (HSP70 359–610 aa, HSP70C) to the Gn and 0.7 kb fragment of the NP (aa1–274-S0.7). C57BL/6 mice were immunized with these recombinant adenoviral vectors. A series of immunological assays determined the immunogenicity of the recombinant adenoviral vectors. The results showed that rAd-GnS0.7-pCAG-HSP70C induced a stronger humoral and cellular immune response than other recombinant adenoviruses (rAd-GnS0.7-pCAG and rAd-GnS0.7) and the HFRS vaccine control. Animal protection experiments showed that rAd-GnS0.7-pCAG-HSP70C was effective at protecting C57BL/6 mice from HTNV infection. The results of the immunological experiments showed that HSP70C lead to enhanced vaccine potency, and suggested significant potential in the development of genetically engineered vaccines against HTNV.

## Introduction

Hantaviruses (HTV) belong to the *Hantavirus* genus of the Bunyaviridae family [1], and are rodent-borne, enveloped RNA viruses consisting of three single-stranded RNA segments, M (medium), S (small) and L (large), which encode the glycoproteins GP, Gn and Gc, nucleocapsid protein (NP), and RNA polymerase, respectively [2]. Hantaviruses cause two febrile illnesses in humans, namely hemorrhagic fever with renal syndrome (HFRS) in the Old World, and hantavirus pulmonary syndrome (HPS) in the New World [1], [3]. At least four HTV species, Hantaan virus (HTNV), Seoul virus (SEOV), Dobrova-Beldrade virus (DOBV), and Puumala virus (PUUV) are associated with HFRS. Andes virus (ANDV) and Sin Nombre virus (SNV) are associated with HPS. The most prevalent and lethal HFRS-associated HTV is HTNV, with more than 100, 000 cases per year, seen mostly in Asia with a case-fatality rate of 10–15% [4], [5]. There are still no effective therapeutic drugs or prophylactic vaccines directed against HFRS until now.

Many investigators have indicated that GP are the constitutive proteins of HTNV, and can elicit an organism to produce neutralizing antibody, and can protect infected animals and humans from lethal HTNV infection [6], [7]. Others have shown that at least two B- and T-cell epitopes exist, and that several neutralization sites existing in Gn [8], [9]. Thus, Gn from HTNV is considered a protective antigen and represents major candidates for genetically engineered HTNV vaccines [10]. Unfortunately, the immunogenicity of Gn is weak, and the antibody titer elicited by Gn is low [8], [11], [12]. The HTNV NP has the strongest immunogenicity among the constitutive proteins. The antibody elicited by NP is long lasting and the titer is high.

It has been shown that NP can induce protection from HTNV infection in experimental animals [13]. Vaccination with DNA encoding HTNV NP has been shown to efficiently elicit a strong NP-specific antibody and CD8^+^ T cell-mediated immune response [14], [15], due in part to there being several B- and T-cell epitopes localized in NP. T-cell epitopes are distributed more randomly [16] while the B-cell epitopes are distributed predominantly closer to the N-terminus [17], [18], and in particular at the 0.7 kb fragment of NP (aa 1–274-S0.7) [17]. Thus, HTNV S0.7 can be used as a component of a protective vaccine. Our previous experiments confirmed that the fusion proteins Gn-S0.7 elicited a relatively good humoral and cellular immune response as compared with the unfused proteins in mice [10], [19].

Since the isolation of HTNV in 1978 [20], several types of inactivated vaccines targeting HFRS have been licensed in China [21]. These vaccines have been produced on the basis of HTNV infected brains of suckling mice, rats or hamsters, and cell culture systems. Large-scale human trials demonstrated a protective efficacy of 94–98% [22]. However, the inactivated vaccine has many shortcomings. One such major shortcoming is the poor immunogenicity to elicit neutralizing antibodies and cell-mediated immunity [23]. Safety is another major obstacle of inactivated vaccine development. As an alternative to the inactivated vaccine approach, candidate HTNV vaccines designed by genetic engineering approaches have been developed. These include recombinant virus vaccines, and vaccines based on naked DNA, recombinant proteins and virus like particles (VLPs) [11], [24], [25], [26]. Despite years of effort, to date there are no vaccines that have been proven to be efficacious against HTNV diseases.

Some strategies aimed at improving the efficacy of genetically engineered vaccines have been studied [22], [27]. One approach is aimed at enhancing vaccine potency combined with adjuvants like Freund’s adjuvant [18], [28], [29], alum [30] or molecular adjuvants [31]. Moreover, HSPs as molecular adjuvants have been used as attractive immunostimulatory components in the development of vaccines. The most important function of HSPs is to serve as molecular chaperones, assisting the correct folding of proteins synthesized *de novo* or denatured by physiological stresses such as heat shock [32]. HSP70 and other HSP family members endogenously bind antigenic peptides in tumor or virus-infected cells. Such HSP70-peptide complexes, formed by fusing antigens to HSP70, are capable of inducing potent antitumor [33], [34], [35], [36] or antiviral immunity [25], [37], [38]. Genetically fusing antigens to HSP70 leads to an enhanced vaccine potency. These investigations have made HSP70 an attractive molecular adjuvant in vaccine development. It has been reported that the HSP70 C-terminal gene (aa 359–610, HSP70C) could play the same role as the HSP70 gene [39]. However, studies showing the effects of the HSP70C gene as a molecular adjuvant aimed at enhancing the potency of DNA vaccines in HTNV vaccine development are currently lacking.

In the current research, cloning the HSP70C gene into the recombinant adenoviral transfer vector GnS0.7-pCAG-pShuttle helped develop a recombinant adenovirus called rAd-GnS0.7- pCAG-HSP70C. The chimeric gene was cloned into the Adeno-X™ viral vector and the recombinant adenoviruses were packaged. The immunological properties of the recombinant adenoviruses in a C57BL/6 mouse model were then studied. Based on the findings of this study, the recombinant adenoviral vector rAd-GnS0.7-pCAG- HSP70C dramatically enhanced the vaccine potency of rAd-GnS0.7-pCAG by augmenting activation of both humoral and cellular immune responses, and could thus protect C57BL/6 mice from HTNV infection. We believe that the rAd-GnS0.7-pCAG-HSP70C vector holds promise as a potential application and novel candidate vaccine to protect against HTNV infection.

## Materials and Methods

### Animals

Adult female C57BL/6J mice, and adult male and female Sprague-Dawley rats weighing 220–250 g, were obtained from the animal research centers of the 4th Military Medical University (Xi’an, China). Mice were housed in isolated and ventilated cages, and the animal work was approved by the Fourth Military Medical University Medical Ethics Committee (Xi’an, China). The approval number was XJYYLL-2012508. The animals were acclimated to the laboratory environment for 5–7 d before use. While in their home cage environment, they were allowed free access to a standard animal diet and tap water. The room was maintained at 20–23°C with a 12 h/12 h light/dark cycle. The animals were deeply anesthetized with inhaled isofluorane (1–3%, or as needed) before all the operations. All efforts were made to minimize animal suffering, reduce the number of animals used, and utilize alternatives to *in vivo* experiments, whenever appropriate or feasible.

### Viruses and Cells

HTNV strains 76–118 were provided from our library. The Adeno-X™ expression system including the pshuttle vector and Adeno-X™ viral DNA was obtained from Clontech (Mountain View, CA, USA). Purified GP was obtained from the Lanzhou Biological Product Academy, and the NP was expressed and purified by our laboratory. The human embryonic kidney (HEK) cell line 293(obtained from the ATCC, Rockville, MD, USA) was used for packaging and propagating of the recombinant adenoviruses, and maintained in Dulbecco’s Modified Eagle’s Medium (DMEM) (Gibco, Grand Island, NY, USA), which was supplemented with 10% fetal bovine serum (FBS) (HyClone, Logan, UT, USA). Vero E6 cells (Vero C1008, ATCC CRL 1586) used for the cellular microculture neutralization test, and B16 murine melanoma cells (B16F10, ATCC) were used for establishing target cells for the CTL assay, and were maintained in RPMI-1640 (Invitrogen, Carlsbad, CA, USA), which was supplemented with 10% fetal calf serum (FCS) (Gibco). All cells were incubated at 37°C in an atmosphere of 5% CO_2_ in 95% air.

### Antibodies

Monoclonal antibodies (mAb) 1A8 (specific to the HTNV NP) and 3G1 (with a high neutralizing Ab activity against HTNV) were prepared in our laboratory [40]. Our laboratory provided Sp2/0 ascites. Mouse anti-HSP70 antibody and other secondary antibodies like FITC-conjugated goat anti-mouse antibody and HRP-conjugated goat anti-mouse antibody were obtained from Sigma (St. Louis, MO, USA).

### Vaccine

The HFRS inactivated vaccine (YOUERJIAN®) used in this study was commercially obtained from Zhejiang Tianyuan Bio-Pharmaceutical Co., Ltd., China, which are provided as a bivalent and purified HFRS vaccine that is derived from a mixture of both HTNV and SEOV.

### Modification to the GnS0.7-pCAG Vector and Packaging of Recombinant Adenovirus

The recombinant plasmid GnS0.7-pCAG, and the recombinant adenoviral constructs rAd-GnS0.7, rAd-GnS0.7-pCAG were developed as described previously [10]. The HSP70C gene was amplified by PCR with the HSP70C up primer (5′-GCGGCCGCATGGAGGTGAAAGACGTTCTGCTGC-3′) and the HSP70C down primer (5′-GCGGCCGCGGTACCTCACTTGG CCTCCCGGCCGTCGTCGT-3′). The HSP70C fragment was inserted into the GnS0.7-pCAG at the *Not*I restriction enzyme sites. The reconstructed vector was named GnS0.7-pCAG- HSP70C. The expression cassette was excised from the reconstructed vector by digesting with PI*-Sce* I and I*-Ceu* I. The Adeno-X™ viral DNA was digested with the same restriction enzymes. The positive recombinant adenoviral DNA was linearized by *Pac* I and transfected into early-passage HEK 293 cells with Lipofectamine 2000 according to the manufacturer’s instructions. The recombinant adenoviral constructs designated as rAd-GnS0.7- pCAG-HSP70C were amplified and purified using the ViraBind™ Adenoviral Purification Kit (Cell Biolabs, San Diego, CA, USA), and their titers were determined using the Adeno-X™ Rapid Titer Kit (Clontech, Mountain View, CA, USA). The adenovirus, which was used as a negative control, and the recombinant adenoviral constructs rAd-GnS0.7, rAd-GnS0.7-pCAG were prepared in the same way. The final preparations were stored at −80°C.

### Identification of the Fusion Protein GnS0.7-HSP70C Expressed in HEK 293 Cells Infected with rAd-GnS0.7-pCAG-HSP70C

To determine the expression of protein S0.7 and HSP70C, infected HEK 293 cells were harvested after 48 h, resuspended in 0.01 M PBS (pH 8.0) and prepared as a monolayer on glass slides by acetone fixation. HEK 293 cell antigen slides were incubated at 37°C for 30 min with mAb 1A8 or anti-HSP70 antibody. FITC-conjugated goat anti-mouse antibody was used as a secondary antibody to demonstrate the positive signal of S0.7 or HSP70C protein expression by fluorescence microscopy.

### Vaccination of Mice with Recombinant Adenovirus

Female C57BL/6 mice were divided into six groups with ten mice for the experimental and control groups, respectively. The experimental groups were immunized with 0.5 ml of 10^8^ ifu/ml recombinant adenovirus per mouse. The control groups were immunized with 10 µl HFRS vaccine, 0.5 ml of 10^8^ ifu/ml adenovirus or 0.5 ml physiological saline per mouse, respectively. All the immunizations were given three times at 2 wk-intervals. Mouse sera were collected individually by retroorbital plexus puncture 10 days after the last immunization. Additionally, splenocytes were isolated for subsequent assays.

### Detection of HTNV-specific Antibodies and Neutralizing Antibody

HTNV NP- and GP-specific antibody titers were determined by an indirect enzyme-linked immunosorbent assay (ELISA). Purified NP or GP was used as coating antigens. Serial dilutions of sera starting at a dilution of 1∶10 were added to the plates and reacted with NP or GP. Anti-NP antibody 1A8 or HFRS patient serum diluted at 1∶100 were used as positive controls. HRP- conjugated anti-mouse antibody was used for detection. The absorbance was measured at a wavelength of 490 nm with a standard ELISA plate reader. The antibody titers were defined as the reciprocal of the serum dilution with the highest positive response.

The cell micro-culture neutralization test was performed on monolayers of Vero E6 cells grown in a 96-well tissue culture plate with the HTNV 76–118 strain. Cells grown in RPMI-1640 medium supplemented with 10% FCS were plated at a density of 2×10^4^ cells well^−1^ 18–24 h before testing. The sera was filtered through 0.22-µm filters and were then two-fold serially diluted from an initial 1∶5 dilution in RPMI-1640 containing 2% FCS, and combined with an equal volume of 100 TCID_50_ HTNV (76–118 strain). After 90 min, the mixture was transferred to monolayers of Vero E6 cells and incubated at 37°C for 9 to 11 days in a 5% CO_2_ incubator. Thereafter, the cells were lysed by three consecutive freeze-thaw cycles. HTNV antigen in the cell lysates was detected by sandwich ELISA. The mAb 1A8 was used as a coating antibody, and HRP-conjugated 1A8 was used as the detecting antibody. The mAb 3G1 and Sp2/0 ascites were used as positive and negative controls respectively. The absorbance was measured at a wavelength of 490 nm using a standard ELISA plate reader. The neutralizing antibody titer was defined as the maximum dilution of serum that inhibited HTNV infection in 50% of the cells.

### Detection of Cytokines Secreted by T Cells

The enzyme-linked immunospot (ELISPOT) assay was used to determine the frequency of responding T cells capable of secreting IFN-γ upon stimulation. Mice were sacrificed and orbital blood samples collected 10 days after the final booster immunization. Spleen cells were purified in lymphocyte separation medium. Freshly isolated splenocytes (1×10^6^ cells in 100 µl) were added into each well of pre-coated IFN-γ microtiter plates (Mabtech, AB, Sweden), and stimulated with purified HTNV GP antigen (10 µg/ml), or the positive mitogenic stimulator concanavalin A (ConA, 4 µg/ml). Splenocytes incubated with 100 µl 2% FCS supplemented DMEM were used as negative or background controls. These plates were incubated at 37°C for 18 h. Development of cytokine ELISPOTs were performed according to the manufacturer’s instructions. Spots were counted using an ELISPOT reader system, and the results were expressed as the mean number of specific IFN-γ spot-forming cells per 1×10^6^ splenocytes. The assay for IL-2, IL-4 and IL-10 secretion were similar to that described for IFN-γ.

### Cytotoxicity Assay

The CytoTox 96™ nonradioactive cytotoxicity assay kit (Promega Co., Madison, WI, USA) was used according to the manufacturer’s instructions to detect the level of specific toxicity in response to the HTNV fusion protein GnS0.7. B16 cells (target cells) transfected with GnS0.7-pCDNA3.1 and screened by G418 were plated in triplicate at 1×10^4^ cells well^−1^ in a volume of 50 µl on 96-well U-bottomed microtitre plates. The splenocytes (effector cells), were prepared as described above and added in a final volume of 50 µl at an effector/target (E/T) ratio of 100∶1, 50∶1 and 20∶1, respectively. Normal splenocytes were added as a negative control. The assay plate included the following as controls: spontaneous LDH release of effector cells (50 µl target cells and 50 µl 5% FCS RPMI-1640), spontaneous LDH release of target cells (50 µl target cells and 50 µl 5% FCS RPMI-1640), target cell maximum LDH release (50 µl target cells, 50 µl 5% FCS RPMI-1640 and 10 µl of the lysis solution), a volume correction control (100 µl 5% FCS RPMI-1640 and 10 µl of the lysis solution), and culture medium background control (100 µl 5% FCS RPMI-1640). The percentage of HTNV GnS0.7-specific lysis was calculated according to the formula: % cytotoxicity = [(*E−S_t_−S_e_*)/(*M−S_t_*)]×100 (E: effector-target co-culture cells LDH release, St: target cell spontaneous LDH release, Se: effector cell spontaneous LDH release, M: target cell maximum LDH release).

### Animal Protection Experiments

Ten days after the final booster immunization, we infected C57BL/6 mice with the HTNV 76–118 strain by intramuscular injection (1×10^5^ pfu/mouse), and detected HTNV specific antigen from the major tissues of C57BL/6 mice, including: cerebrum, heart, liver, spleen, lung and kidney by ELISA after 3 days. Tissue samples were weighed and diluted in PBS to be the 10% (g/ml) tissue suspensions, then freeze thawing (−80/37°C) three times after grinded. The samples were centrifugated at 12,000 × rpm for 30 min at 4°C, and the supernatants were collected. HTNV antigens in the supernatants were detected by sandwich ELISA. The mAb 1A8 was used as a coating antibody, and HRP-conjugated 1A8 was used as the detecting antibody. The normal tissue supernants were used as negative controls. The absorbance was measured at a wavelength of 490 nm with a standard ELISA plate reader. An OD490 value exceeding the 2.1 fold of negative controls was considered as positive and it meant that the HTNV specific antigen could be detected from the sample.

### Histological Analysis

Male and female Sprague Dawley rats were divided into two groups with six rats (three male and three female) for the experimental and control groups, respectively. The experimental group was immunized with 0.5 ml of 1.5×10^9^ ifu/ml rAd-GnS0.7-pCAG-HSP70C per rat, which was 50 times the usual normal dose given to rat models. The control group was a group of rats that were left untreated like normal controls. All of the immunizations were given three times at 2 wk intervals. The heart and some other organs of the treated and untreated rats were collected individually 10 days after the last immunization. Tissue samples were embedded in blocks of paraffin and cut with a microtome into 5 µm sections, which were subsequently mounted onto glass slides and stained with hematoxylin-eosin (HE).

### Statistical Analysis

All data were expressed as the mean ± the standard error of mean (SEM) and are representative of at least three independent experiments. The Student’s *t*-test was used to determine significant differences between paired experimental and control groups. One-way ANOVA was used to determine statistically significant differences among the experimental groups. A *p* value of 0.05 or less was considered significant.

## Results

### Identification of the Construction and Expression of rAd-GnS0.7-pCAG-HSP70C

**Figure 1:**
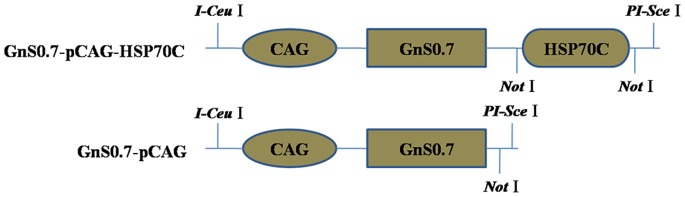
Showing the construction strategy of the GnS0.7-pCAG-HSP70C expression plasmid. The recombinant plasmid GnS0.7-pCAG was previously constructed in our laboratory. The HSP70C sequence was introduced into the C-terminal of GnS0.7-pCAG. The locations of the restriction enzyme sites are indicated.

**Figure 2:**
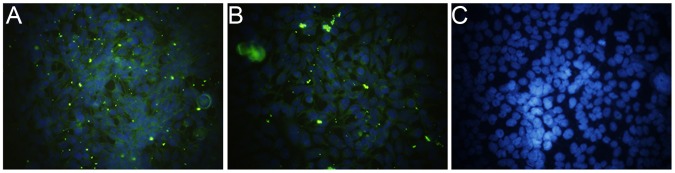
Immunofluoresence assay of the S0.7 and HSP70C proteins that were expressed in HEK 293 cells following transduction with rAd-GnS0.7-pCAG-HSP70C. HEK 293 cell antigen slides were prepared for detection of S0.7 and HSP70C. Mouse monoclonal antibody 1A8 or mouse anti-HSP70 antibody was used respectively to detect S0.7 or HSP70C. FITC-conjugated goat anti-mouse antibody was used to stain for positive fluorescence signals. Normal HEK 293 cells were used as negative controls. (A), S0.7 protein as detected with 1A8; (B), HSP70C protein as detected with anti-HSP70 antibody; and (C) normal HEK 293 cells as detected with a mixture of the above monoclonal antibodies.

Briefly, the HSP70C sequence was cloned into the C-terminal of GnS0.7-pCAG (Fig. 1). The accuracy of cloning in each of these constructs and the packaging of recombinant adenoviral constructs was confirmed by restriction enzyme and polymerase chain reaction (PCR) analyses. After purification, the recombinant adenoviral construct rAd-GnS0.7-pCAG- HSP70C was concentrated to 10^10^ ifu/ml. HEK 293 cells were infected with rAd-GnS0.7- pCAG-HSP70C and were then examined by immunofluorescence assay (IFA). Positive fluorescence was observed in cells 48 h after infection (Fig. 2A and B).

### Induction of Humoral Immune Responses after Immunization with Recombinant Adenoviruses Carrying GnS0.7

**Figure 3:**
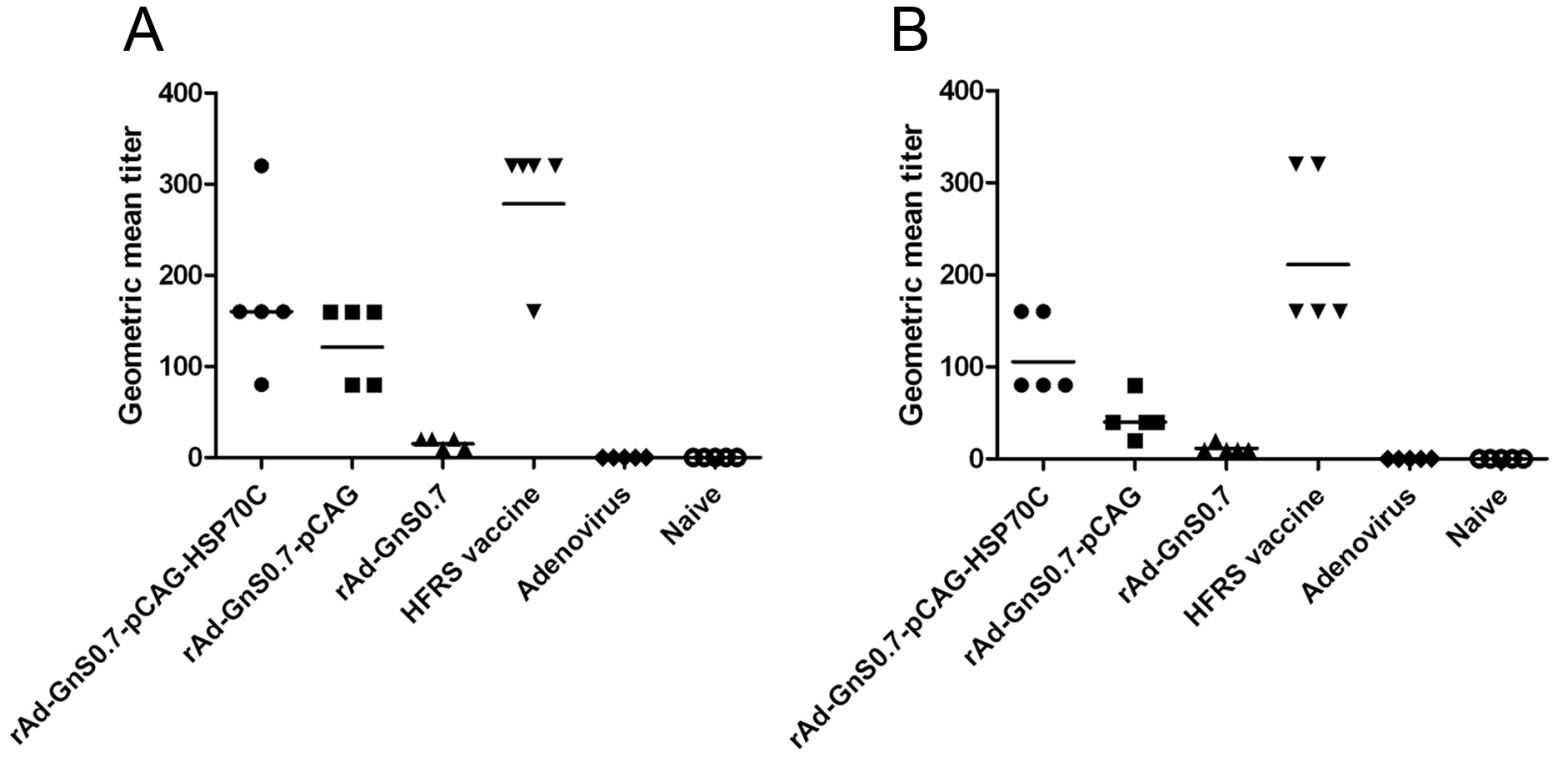
Humoral immune responses in C57BL/6 mice elicited by recombinant adenoviral vectors containing GnS0.7 and HFRS. (A), HTNV NP specific antibody production. (B), HTNV GP specific antibody detection. The geometric mean titers of mice immunized with rAd-GnS0.7-pCAG-HSP70C against NP and GP were the highest among all vector groups.

The geometric mean titers (GMT) of mice immunized with rAd-GnS0.7-pCAG-HSP70C against NP and GP were the highest among all the different recombinant adenoviral groups (1∶160 and 1∶105.6, respectively). The GMT of mice immunized with the HFRS vaccine against NP and GP were 1∶278.6 and 1∶211.1, respectively (Fig. 3A and B). The results from the adenoviral and naive groups were all negative. These results suggest that the specific antibody titers against NP and GP in mice immunized with rAd-GnS0.7-pCAG- HSP70C were higher than any other recombinant adenoviral group but less than the HFRS vaccine group.

**Figure 4:**
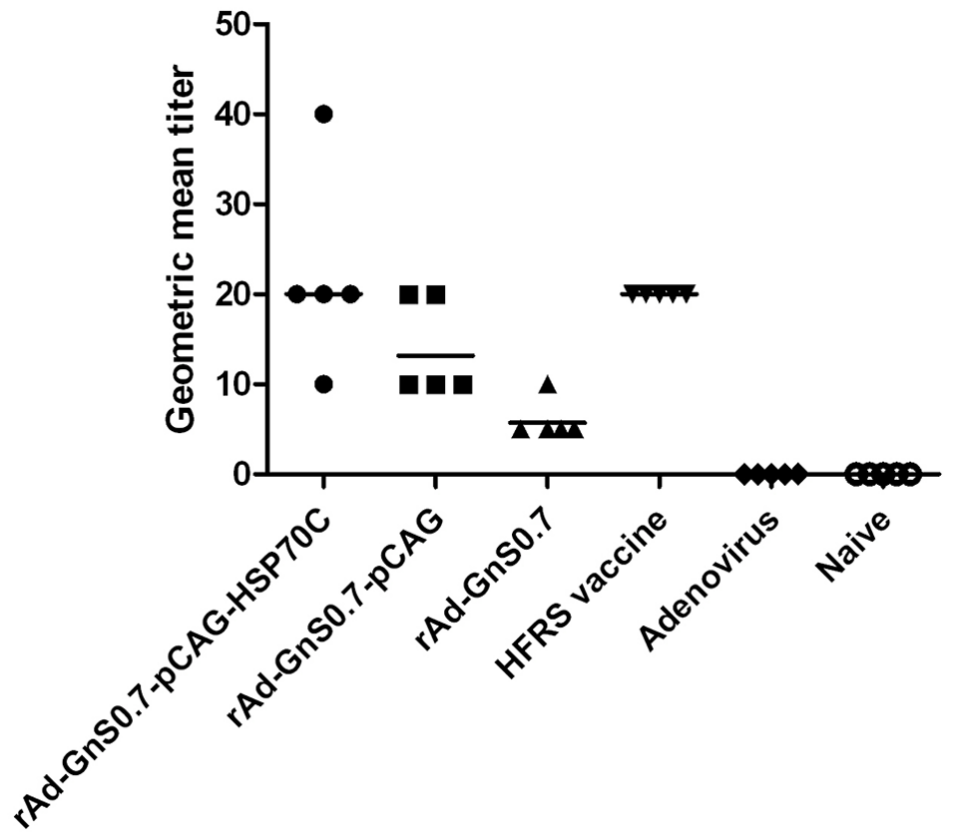
Neutralizing antibody responses against the HTNV 76–118 strain in C57BL/6 mice elicited by recombinant adenoviral constructs containing GnS0.7 and HFRS. The geometric mean titer of neutralizing antibody in C57BL/6 mice immunized with rAd-GnS0.7-pCAG- HSP70C was the highest among all of the different vector groups.

The neutralizing antibody titers of mice immunized with rAd-GnS0.7-pCAG-HSP70C could be up to 40, and the GMT (1∶20) of neutralizing antibody was the highest among all the different recombinant adenoviral groups. The GMT of neutralizing antibody in C57BL/6 mice that were immunized with the HFRS vaccine could be up to 20. The sera from the adenoviral and naive groups showed no neutralizing antibody activity. These results suggested that immunization with rAd-GnS0.7-pCAG-HSP70C could induce neutralizing antibodies in mice against HTNV that was comparable to the current HFRS inactivated vaccine (Fig. 4).

### Induction of Cellular Immune Responses after Immunization with Recombinant Adenoviruses Carrying GnS0.7

**Figure 5:**
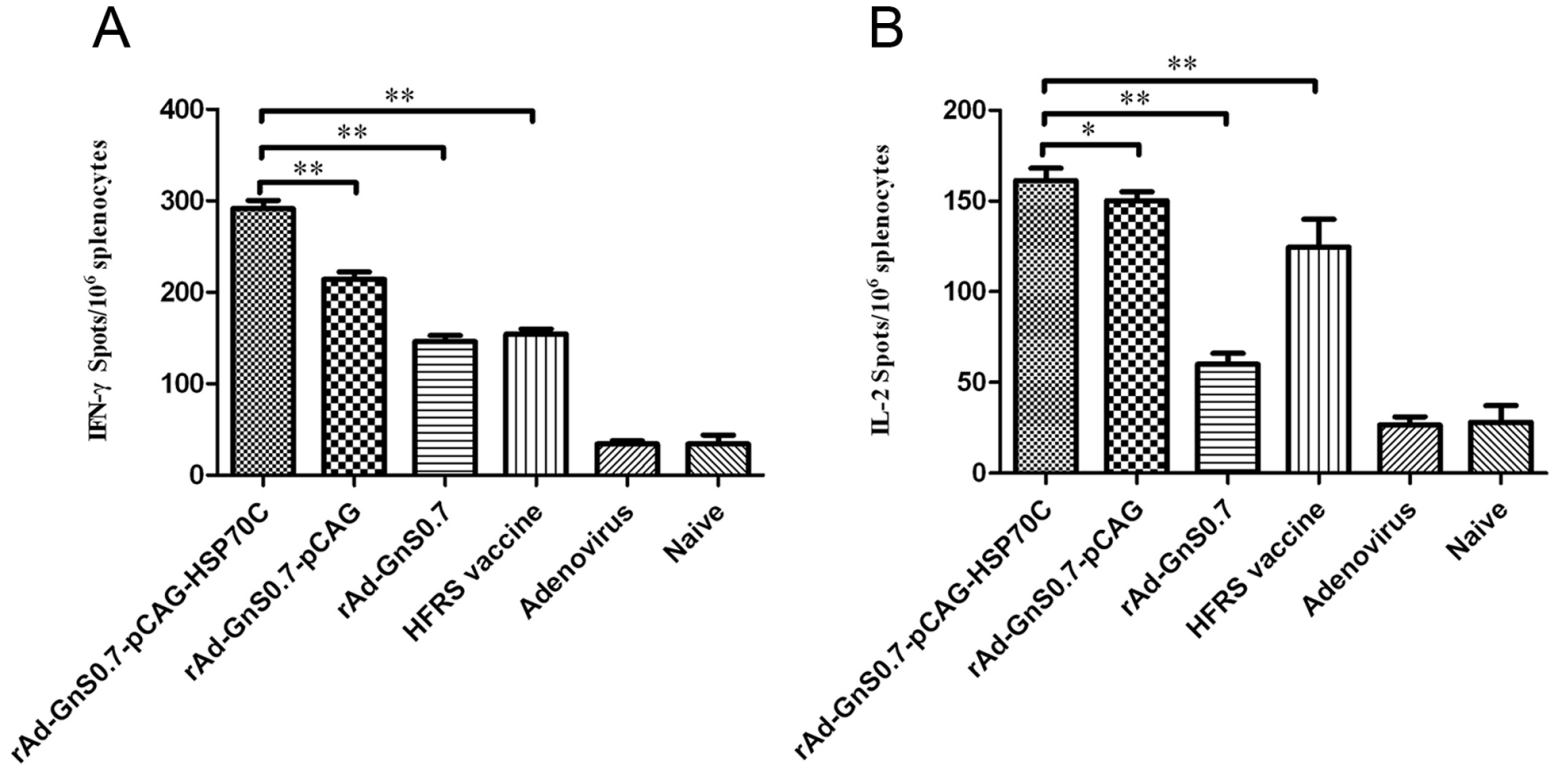
(A) ELISPOT analysis of IFN-γ secretion by splenocytes. All experimental groups could generate IFN-γ (A), of which the rAd-G1S0.7-pCAG-HSP70C construct provoked higher levels of IFN-γ secretion than the other constructs (^**^p<0.01). (B), ELISPOT analysis of IL-2 secretion by splenocytes. All experimental groups generated IL-2, of which the rAd-G1S0.7- pCAG-HSP70C construct induced higher levels of IL-2 secretion as compared the others (^**^p<0.01, ^*^p<0.05). The results are expressed as the mean value ± SEM of three independent experiments.

The frequencies of CD4^+^ T cells secreting IFN-γ, IL-2, IL-4 and IL-10 from the spleens of immunized mice were determined by the ELISPOT assay. The recombinant adenoviruses containing GnS0.7 induced an effective IFN-γ and IL-2 response, and of these, the group immunized with the rAd-GnS0.7-pCAG-HSP70C had a significantly higher response than the other adenoviral and naive groups that induced only negligible IFN-γ and IL-2 responses (Fig. 5A and B). The IL-4 and IL-10 levels did not change remarkably for any of the immunization groups.

**Figure 6:**
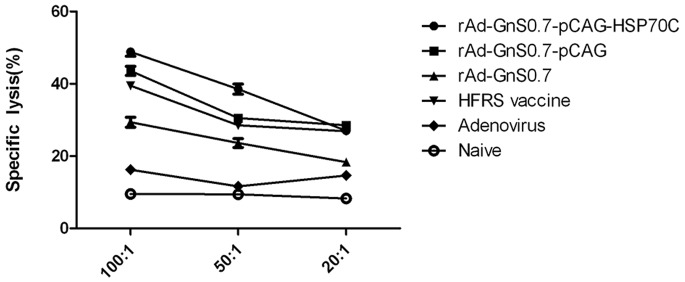
HTNV GnS0.7-specific cytotoxicity against B16-GnS0.7 cells by different recombinant adenoviral constructs. Mice were immunized three times at 2-week intervals with different recombinant adenoviral constructs containing GnS0.7. The results are expressed as the mean value ± SEM of three independent experiments.

LDH release from HTNV GnS0.7-expressing B16 cells that were targeted by vaccination-activated splenocytes was measured using a Cytotox 96 non-radioactive cytotoxicity assay kit. The cytotoxicity of splenocytes from mice immunized with recombinant adenoviruses containing GnS0.7 was enhanced in accordance with the E/T ratio, which was the most significant at the ratio of 100∶1. Among the experimental groups, splenocytes from mice immunized with the rAd-GnS0.7-pCAG- HSP70C showed the highest specific cytotoxic activity than any of the other groups at E/T ratios of 100∶1 and 50∶1 (*p*<0.05). Additionally, it was even higher than the HFRS vaccine group (*p*<0.01), but was found less effective than either the rAd-GnS0.7-pCAG or the HFRS vaccine at an E/T ratio of 20∶1 (*p*<0.05). By contrast, the non-specific cytotoxicity against B16-GnS0.7 cells of control mice in either adenoviral or naïve groups was very weak at E/T ratios of 100∶1, 50∶1 and 20∶1, respectively (Fig. 6).

### Evaluation of Protective Immunity Induced by the Recombinant Adenoviruses Containing GnS0.7 for Preventing HTNV Challenge in Mice

**Figure 7:**
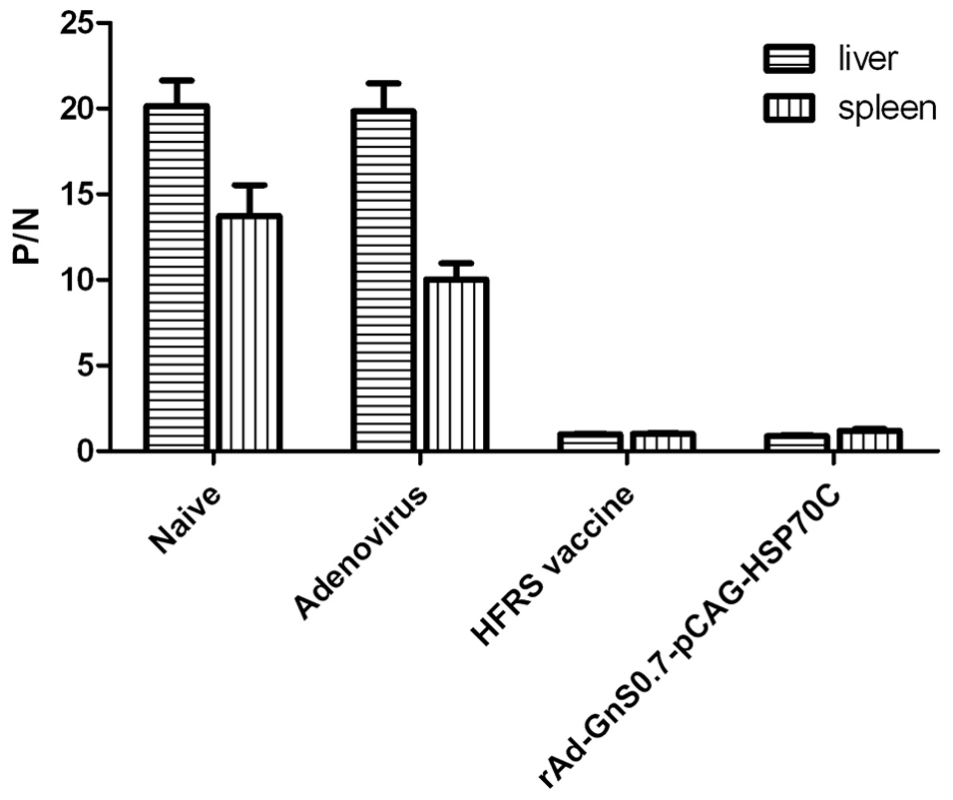
Detection of HTNV specific antigen from both the livers and spleens of C57BL/6 mice. The HTNV specific antigen was detected in the livers and spleens of C57BL/6 mice both ADV and naïve groups. Mice immunized with rAd-G1S0.7- pCAG-HSP70C and the HFRS vaccine remained negative for HTNV-specific antigen. The results are expressed as the mean value ± SEM of four independent experiments.

To assess protective immunity, mice were challenged with HTNV at 10 days after the final booster immunization. We found that the HTNV specific antigen was detected in the livers and spleens of C57BL/6 mice in both the adenoviral (19.841 and 10.026, respectively), and naïve groups (20.159 and 13.740, respectively). However HTNV specific antigen could not be detected from the rAd-GnS0.7- pCAG-HSP70C (0.891 and 1.194, respectively) or the HFRS vaccine groups (0.998 and 1.011, respectively, Fig. 7), and there was no obvious differences in HTNV antigen detection between them (*p*>0.05). HTNV specific antigen could not be detected from other tissues of C57BL/6 mice in all of the groups (data not shown). These results indicated that immunization with rAd-GnS0.7-pCAG-HSP70C and the HFRS vaccine conferred protective immunity in C57BL/6 mice.

### Histopathological Analysis by HE Staining

**Figure 8:**
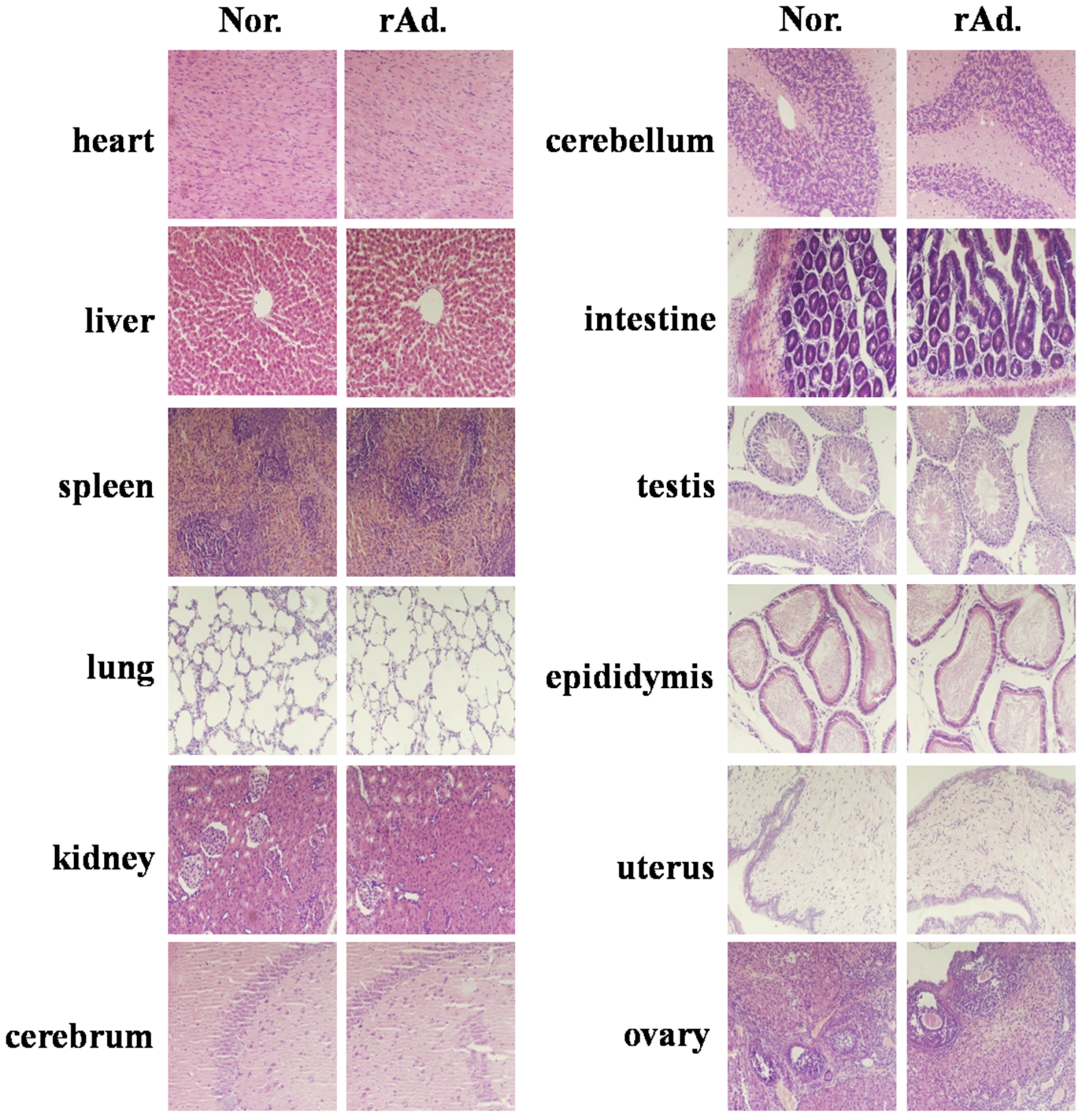
Sections of different tissues from rAd-GnS0.7-pCAG-HSP70C and normal control groups were HE stained. rAd represents the rAd-GnS0.7-pCAG-HSP70C group, and Nor represents the normal control group. HE staining showed that no significant alterations were found between the rAd-GnS0.7- pCAG-HSP70C group and the normal control.

The results showed that no significant alterations were found in the tissues from the rAd-GnS0.7-pCAG-HSP70C group and normal control (Fig. 8). We also collected other tissues from the rats for HE staining such as the spinal cord, pancreas, adrenal gland, intestinal gland, and other tissues as necessary and found that there were no differences (data not shown). These results indicated that immunization with the rAd-GnS0.7-pCAG-HSP70C construct exhibited no pathological damage in the rats.

## Discussion

In this study, we demonstrate that vaccination with rAd-GnS0.7-pCAG-HSP70C sufficiently elicited both humoral and cellular immune responses in mice. The rAd- GnS0.7-pCAG-HSP70C construct elicited specific anti-NP/GP and neutralizing antibodies, and resulted in higher secretion of NP-specific IFN-γ and IL-2 and more potent activation of GnS0.7-specific CTL responses than that found for the HFRS vaccine and other recombinant adenoviruses. Moreover the rAd-GnS0.7-pCAG-HSP70C construct, protected mice from HTNV infection.

Many expression systems have been developed for genetically engineering vaccines of HTNV like the yeast expression system [41], *E. coli* expression system [37], mammalian expression system [42], insect virus expression system [19], the adenovirus expression system [10], and others. In many expression systems, Ad5-based vectors have been extensively applied because they permit the introduction of relatively large DNA sequences, are safe to use and do not integrate into the host genome, are easy to construct and propagate, and can effectively present antigen and induce humoral and cellular immune responses. When different vectors expressing the same antigen were compared, the Ad5-based vectors proved particularly immunogenic, notably with regard to the induction of antigen-specific CD8^+^ T cells [43]. In our previous studies, different systems were examined for expression of the GP and/or NP of HTNV, and immunization of mice with the expression proteins. In these studies, protective immunity that was provided by expression of the proteins using the adenoviral system was more potent [10], [19], [23]. These observations are clearly advantageous and argue in favor of using the adenoviral vector system as an excellent choice in vaccine constructs.

The results of the ELISA and cell micro-culture neutralization tests showed that the rAd-GnS0.7-pCAG-HSP70C construct had some advantages over other recombinant adeno- viral constructs in terms of eliciting a humoral response against HTNV via GnS0.7. In addition, the increased efficacy of the rAd-GnS0.7-pCAG-HSP70C construct was due to its binding with HSP70C. That means that since HSP70C functions as a molecular chaperone and an immune adjuvant, successfully assisted the presentation of the fusion protein GnS0.7 to antigen presenting immune effector cells, and elicited higher specific antibody and neutralizing antibody titers. HSP70C also assisted enhancing the efficacy of cellular immune responses. In our study, the IFN-γ and IL-2 released after immunization with rAd-GnS0.7- pCAG-HSP70C were higher than other recombinant adenoviral constructs and HFRS vaccine groups. By contrast, the secretion of both IL-4 and IL-10 did not change remarkably for all immunization groups. We know that CD4^+^ T cells can differentiated into Th1 phenotype and Th2 phenotype. The Th1 phenotype mainly secretes IFN-γ and IL-2 and which enhance proliferation of CD8^+^ T cells. The Th2 phenotype mainly secretes IL-4 and IL-10 which enhance proliferation and activation of B cells. Polarizing CD4^+^ T cells to a Th1 phenotype generally results in inhibition to a Th2 phenotype and vice versa. This result suggests that Th1 phenotype cells might play a more important role in mice immunized with modified recombinant adenoviral constructs, which were useful for the proliferation, differentiation, and maturation of Tc cells, and were capable of promoting cell-mediated immune responses. Then the results from Cytotoxicity assay confirmed this conclusion.

It has been previously found that vaccination with recombinant HTV NP might require an adjuvant that induces a Th1-type immune response [29], and HSPs may directly active T cells both *in vivo* and *in vitro* in an antigen-independent mechanism that induces the secretion of both IFN-γ and IL-2 in the absence of antigenic peptides [44]. The increase in Th1-type immune responseiveness is consistent with the significantly higher levels of both IFN-γ and IL-2 production after rAd-GnS0.7-pCAG-HSP70C immunization than with other recombinant adenoviral constructs and HFRS vaccine groups. Thus, these observations confirmed that HSP70C modulated the immune response towards the induction of strong cellular responses with a dominant Th-1 cytokine profile.

The HSP70 gene family is similar to the MHC gene, and the HSP70 protein-binding motif is structurally similar to MHC-I molecules. When HSP70-peptide complexes are taken up by antigen-presenting cells (APCs), such as dendritic cells (DCs) [45], [46], this pathway is recognized as the major mechanism that enables coupled polypeptide or protein partners to gain entry into the MHC-I presentation pathway and thus stimulate production of CD8+ CTLs [44], [47]. Thus, HSP70C is capable of promoting antigen presentation by the MHC-I pathway, thereby stimulating the host system to generate strong cytotoxic protective immune effects. In our study, the specific cytotoxic effects of the rAd-GnS0.7-pCAG-HSP70C construct were more potent than other recombinant adenoviral constructs and HFRS vaccine groups at the E/T ratio of 100∶1 and 50∶1, observations that were concordant with the views expressed above. In addition, HSP70C assisted the rAd-GnS0.7-pCAG-HSP70C construct to induce more complete and effective immune responses than did the other recombinant adenoviral constructs and HFRS vaccine groups.

However, we also found that the titers of mice immunized with the inactivated HFRS vaccine against NP and GP were higher than the titers of mice immunized with the rAd-GnS0.7-pCAG-HSP70C construct. The reason for this is currently unclear, but we suspect that the HFRS vaccine contains the complete GP and NP of HTNV, which mean that it contains more antigenic sites than the rAd-GnS0.7-pCAG-HSP70C construct. As we have said that Th1 phenotype cells might play a more important role in mice immunized with modified recombinant adenoviral constructs and resulted in inhibition of Th2 phenotype. The proliferation and activation of B cells were affected and the secretion of antibodies were reduced. As a consequence, the inactivated HFRS vaccine can more easily induce the host to produce higher titers of both anti-GP and anti-NP antibodies. Conversely, the neutralizing antibody titer of mice immunized with the rAd-GnS0.7-pCAG-HSP70C construct could be up to 40 times higher than the HFRS vaccine. That said, we should not ignore the potential capacity of inactivated vaccines to provide protection from HTNV. Several possibilities may be taken into account for the differences seen between rAd-GnS0.7-pCAG-HSP70C and inactivated vaccine constructs with regard the neutralizing antibody titer: i) The inactivated vaccine used in this study without adjuvant might assist the inactivated vaccine to induce neutralizing antibody more effectively; ii) There is general acceptance that neutralizing antibodies should be a central goal of anti-HTNV vaccination, while on the other hand, long-lasting, high-titer neutralizing antibody responses may not be necessarily critical for HTNV vaccine to confer an efficacious protective immune response in the population [48].

In the process of HFRS vaccine research, animal protection experiments represent an important indicator of the effectiveness of the vaccine, and selection of the most appropriate animal model is key to animal protection experiments. The development of animal models of HFRS has been an intense area of research. Currently no animal model exists that reflects disease manifestations of severe HFRS. Non-human primates often provide good models for studying hemorrhagic fever viruses, and therefore have been assessed as potential models for HFRS, though with limited success [49], [50]. Several small laboratory animals have been experimentally infected with HTNV species resulting in the characterization of traditional infection and/or acute disease models. Perhaps the most successful of these are infections of newborn (suckling) [51], [52], [53] and juvenile mice [54], [55] with HTNV which, depending on the virus strain, and route of infection utilized, usually results in lethal disease. However, we also know that newborn and juvenile mice are not immunocompetent, and not suitable for animal protection experiment of vaccines. In our study, we found that 3 days after infection with HTNV 76–118 by intramuscular injection, the specific antigen could be detected in the livers and spleens of C57BL/6 mice in both adenoviral and naïve groups, but not in rAd-GnS0.7-pCAG-HSP70C or HFRS vaccine groups. These observations provided some information that in mice immunized with the rAd-GnS0.7-pCAG-HSP70C construct, there was protection against HTNV 76–118 infection, and according to our observations, we might identify a new approach for animal protection experiments for the HTNV vaccine.

In summary, the results presented in the current study have demonstrated detection of relevant indicators of the host immune response to rAd-GnS0.7-pCAG-HSP70C, and the potential application of rAd-GnS0.7-pCAG-HSP70C as a new type of HTNV vaccine. However, much more work will be needed before a firm conclusion can be made with regard future perspectives of this vaccine and its potential clinical use.
